# Association between elite swimmers’ force production and 100 m front crawl inter-lap pacing and kinematics

**DOI:** 10.3389/fspor.2023.1205800

**Published:** 2023-05-26

**Authors:** Mário J. Costa, Catarina C. Santos, Francisco Ferreira, Raul Arellano, J. Paulo Vilas-Boas, Ricardo J. Fernandes

**Affiliations:** ^1^ Centre of Research, Education, Innovation, and Intervention in Sport (CIFI2D), Faculty of Sport, University of Porto, Porto, Portugal; ^2^ Porto Biomechanics Laboratory (LABIOMEP-UP), University of Porto, Porto, Portugal; ^3^ Department of Sport Sciences, University of Beira Interior, Covilhã, Portugal; ^4^ Research Center in Sports Sciences, Health Sciences and Human Development (CIDESD), Covilhã, Portugal; ^5^ Aquatics Lab, Department of Physical Education and Sports, Faculty of Sport Sciences, University of Granada, Granada, Spain

**Keywords:** tethered swimming, training, velocity, peak force, performance

## Abstract

The present study aimed to analyse the associations between force production and 100 m front crawl inter-lap pacing and kinematics. Eleven elite male swimmers performed a 100 m front crawl maximal effort to collect 50 m lap time (T_50_, s) and velocity (v, m·s^−1^) for pacing, stroke rate (SR), stroke length (SL) and stroke index (SI) as kinematic variables. A 30 s tethered effort allowed to determine the peak (F_peak_) and mean force (F_mean_) as force production variables. The relative change (Δ) between 50 m laps was also calculated for all measures. A paired sample *t*-test was used to check differences between laps and Pearson correlation coefficients allowed to quantify the associations between force and remaining variables. The T_50_ increased from the first to the second lap (ΔT_50_ = 10.61%, *p* < 0.01, d = 2.68), while v (Δv = −5.92%, *p* < 0.01, d = 1.53), SR (*Δ*SR = −6.61%, *p* < 0.01, d = 0.45) and SI (ΔSI = −4.92%, *p* = 0.02, d = 0.45) decreased. SL remained unchanged between laps (ΔSL = 1.07%, *p* = 0.66, d = 0.08). No associations were found between force production and most of Δ, with the only exception being the reasonable good association between F_peak_ and Δv (r = 0.62, *p* = 0.04). Although both pacing and kinematics fall from the first to the second sections of a 100 m front-crawl effort, the swimmers who exhibit higher F_peak_ show a more stable front crawl v between both 50 m laps.

## 1. Introduction

The way how a swimmer behaves within a specific effort or event is still a topic of debate among swimming coaches and researchers. For great performances, the swimmer needs to exhibit the capacity to move forward by effectively applying force in the water, but also show the appropriate body shape to reduce drag forces acting in the opposite direction of the displacement (1, 2). This means that force production and technique are topics of great importance for the training process and should be part of a regular and systematic assessment in swimming squads.

Nowadays, the measurement of force production in swimming can be performed with experimental methods where the tethered swimming is framed (3). Tethered swimming has been proposed as a reliable method to assess the swimmer's in-water force based on peak and mean force values (4). Previous studies denoted positive relationships between peak or mean force values and the 50, 100 and 200 m front crawl velocities (5, 6). Although there is a good rationale on how force production is associated with short or middle-distance performance, there is limited information about its impact according to inter-lap changes.

The inter-lap assessment is an approach that allows understanding the swimmers’ variance in a given variable during a specific effort (7), and it proves to be crucial in closely matched swimmers when appropriate pace and kinematics maintenance can determine the difference between winning or losing (8). Successful swimmers can keep their velocity more constant and stable throughout a single race when compared to their less skilled counterparts (9). While explanations for this success may rely on a high stroke length and stroke index, with both parameters linked to swimming efficiency (10), the increases in stroke rate associated with a slight decrease in stroke length should not be considered ineffective (11, 12). Although for kinematics this seems to be an enlightened topic, the same does not happen for the force production.

It is well documented that any prolonged muscle activity leads to a reduction in mechanical muscle power affecting force production (13). At least in swimming, the changes in technique during an exhaustive effort are explained, in part, by the fatigue of the upper limbs muscles (14). As the great amount of propulsion at the front crawl comes from the upper limbs actions (15), it can be questioned if inter-lap changes in kinematics are due to finer motor adaptations or changes in force production that ultimately can affect velocity. High-skilled swimmers tend to be more adaptive by showing a broader functional adaptation in force parameters when different swimming paces were used (16).

However, the available studies on the topic just established associations between the swimmers’ force and the overall race pace discarding any inter-lap changes. It remains unanswered if the swimmers that show a greater force in tethered swimming are more prone to keep their pace and kinematics during a 100 m effort. This kind of knowledge could help coaches and researchers to define inter-lap pacing and performance behaviours based on force production assessment. The present study aimed to analyse the associations between elite swimmers’ force production and the inter-lap pacing and kinematics during a 100 m front crawl effort. It was hypothesized that would exist a positive association between force and the ability to maintain pacing and kinematics.

## 2. Methods

### 2.1. Participants

Eleven male swimmers from the same squad (18.3 ± 2.8 years of age, 74.8 ± 8.6 kg of body mass, 1.82 ± 0.08 m of height) volunteered to participate in this study. The inclusion criteria for the participants were: (i) being front crawl specialists; (ii) framed as elite level (17); (iii) practicing more than seven training sessions per week; and (iv) not having suffered any injuries in the past six months. The level of the swimmers is given by 704 ± 67 World Aquatics points in the 100 m front crawl event (long course pool) considering their personal best in the past twelve months. The swimmers were informed about the benefits and experimental risks before signing a written informed consent form. All procedures were in accordance with the Declaration of Helsinki and approved by the local Ethics Committee.

### 2.2. Design and experimental procedures

Participants attended two experimental sessions in the morning on different days 48 h apart. They were asked to abstain from intense exercise in the two days before the tests to avoid data bias due to fatigue. The first session was to collect anthropometrics and to simulate a 100 m front crawl effort. The swimmers arrived in a well-rested condition for anthropometric measurements wearing only a textile swimsuit and a cap. Height and body mass were measured with a digital stadiometer (SECA, 242, Hamburg, Germany) and a scale (TANITA, BC-730, Amsterdam, Netherlands), respectively. After, swimmers were instructed to perform a standardized warm-up at low intensity (400 m soft swim, 100 m pull, 100 m kick, 4 × 50 m at increasing speed and 200 m recovery) before the in-water experimental testing. A 10 min recovery was allowed to avoid any fatigue effect before performing a 100 m maximal effort at front crawl. The in-water testing was carried out in a 50 m indoor swimming pool (water temperature of 27.5°C and relative humidity of 60%) and the race simulation started from the starting block after an official auditory stimulus.

The second session was to measure in-water forces through the tethered swimming method. Swimmers repeated the warm-up routine as in the first testing session. Then, a 30 s tethered (full body) swimming was performed at maximal intensity. The swimmers used a belt around their waist and remained connected to a load cell system (Globus™, Codognè, Italy) using a steel cable (3.5 m length) attached to the starting block. The calibration of the load cell was verified before the test by using specific loads, as reported elsewhere (18). To avoid the inertial effect, participants began the test by swimming for 5 s at low intensity before starting the 30 s maximum effort. A stopwatch (FINIS 3 × 300, Finis Inc., USA) and an auditory signal were used to control the start and the end of the test. The swimmers were already familiar with the tethered swimming protocol, not needing to account for adaptation issues, and their normal breathing pattern in sprint events was encouraged to be used.

### 2.3. Data collection

The swimming performance was determined as the time spent to cover the 100 m and registered by the chrono set-up used in official competitions. The 50 m lap time (T_50_, in s) and velocity (v, in m·s^−1^) were used for pacing determination. The v and kinematic variables were assessed between the 15th and the 35th marks in both 50 m laps to exclude any starting or turning effects. The stroke rate (in Hz) was manually assessed by a certified coach with a chrono-frequency meter (FINIS 3 × 300, Finis Inc., USA) from three consecutive cycles. The stroke length (in m) was then estimated (stroke length = velocity / stroke rate) as reported elsewhere (19). The stroke index (in m^2^·s^−1^) was computed as stroke index = velocity · stroke length (10). The relative change (Δ, in %) between 50 m laps was also calculated for all measures adapting the equation [(2nd50m—1st50m)/(1st50m) · 100] that was previously used for pacing variability (20).

In the tethered swimming, the data was acquired with a sampling frequency of 100 Hz. The load cell was connected by a cable to a Globus Ergometer data acquisition system that exported the data in ASCII format to a PC. Data were then imported into a signal-processing software (AcqKnowledge v.3.7.3, Biopac Systems, Santa Barbara, CA, USA) and the signal was handled with a 5 Hz cut-off low-pass fourth-order Butterworth filter. The peak force (in N) was defined as the highest value obtained from the individual force-time curves of three consecutive cycles after the beginning of the test. The first two cycles were discarded due to the inertial effect as the swimmer remained stationary. The mean force (in N) through the overall 30 s period was also calculated. An angle correction of 6° was considered for computing the horizontal component of force (21).

### 2.4. Statistical analysis

The normality and homoscedasticity of the data were verified by the Shapiro-Wilk and Levene tests, respectively. Mean and standard deviation were obtained for standard descriptive analysis. A paired sample t-test was used to check differences between 50 m laps in all variables. Cohen's d was selected as effect size and interpreted as trivial if d < 0.2, medium if 0.2 > d < 0.5, and large if d ≥ 0.5 (22). Pearson correlation coefficients (r) were determined between force and remaining variables, being thereafter interpreted as low if r < 0.30, moderate if 0.30 ≥ r < 0.60, and reasonably good if r ≥ 0.60 (23). Scatter plots with individual values and 95% confidence limits were computed to illustrate associations. All statistical analyses were performed using the SPSS software (v.27, IBM, SPSS Inc., Chicago, IL, USA). The statistical significance was set at *p* ≤ 0.05.

## 3. Results

**Figure 1:**
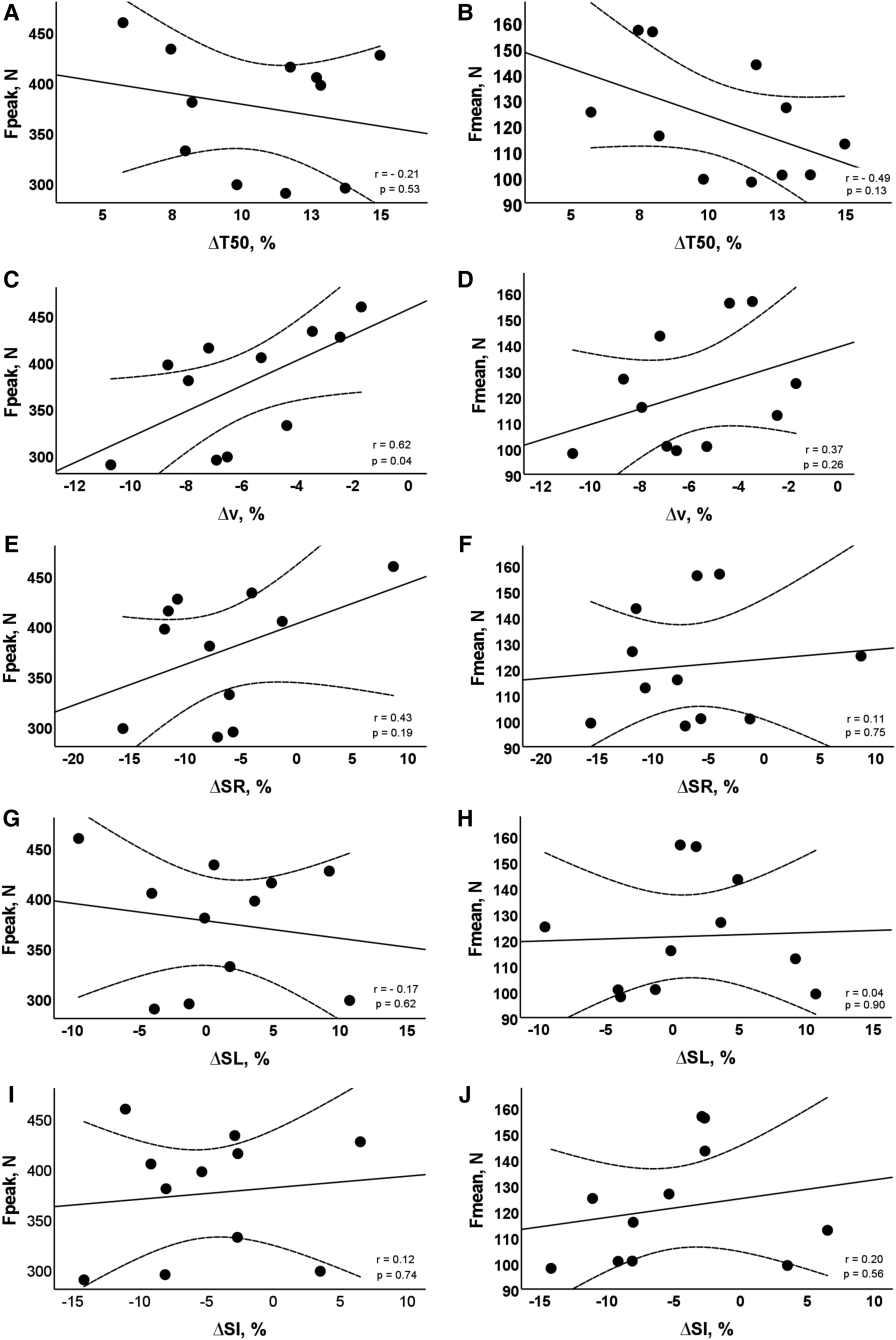
Scatter diagrams about the association between force variables and the relative change in pacing and kinematic measures. Individual values and 95% confidence limits are presented. Fpeak, peak force; Fmean, mean force; ΔT50, relative change in 50 m time; Δv, relative change in velocity; ΔSR, relative change in stroke rate; ΔSL, relative change in stroke length; ΔSI, relative change in stroke index.

The mean performance time of the 100 m effort was 56.18 ± 1.96 s. The comparison of temporal and kinematic measures between both 50 m laps is shown in Table 1. While the T_50_ increased from the first to the second 50 m lap, the velocity, stroke rate and stroke index showed a decrease. The stroke length was the single variable that remained unchanged between laps. The force data retrieved during the 30 s tethered swimming test showed a peak force of 375.19 ± 61.31 N and a mean force of 121.35 ± 22.29 N. The associations between force variables and the relative change in temporal and kinematic measures are shown in Figure 1. No associations were found between force production and most of *Δ*. The only exception was the reasonable good association between peak force and Δv (panel C).

**Table 1:** Comparison of pacing and kinematic variables between the 1st and the 2nd 50 m laps during the 100 m front crawl effort.

Variable	1st Lap	2nd Lap	*p*	*d*	Δ
Time at 50 m (s)	26.67 ± 0.73	29.51 ± 1.31	<0.01	2.68	10.61
Velocity (m·s^−1^)	1.76 ± 0.07	1.66 ± 0.06	<0.01	1.53	−5.92
Stroke rate (Hz)	0.84 ± 0.12	0.79 ± 0.10	<0.01	0.45	−6.61
Stroke length (m)	2.12 ± 0.25	2.14 ± 0.23	0.66	0.08	1.07
Stroke index (m^2^·s^−1^)	3.74 ± 0.43	3.55 ± 0.42	0.02	0.45	−4.92

## 4. Discussion

The present study aimed to analyse the associations between elite swimmers’ force production and 100 m front crawl inter-lap pacing and kinematics. Although both temporal and kinematic variables fall from the first to the second part of the effort, the swimmers who exhibited higher peak forces were the ones who showed a more stable swimming velocity between both 50 m laps. Since our hypothesis was partially confirmed, coaches can rely on peak force values to monitor their swimmers’ pacing potential.

Most of the assessed variables got worse from the first to the second 50 m laps, which is in agreement with previous studies on pacing and kinematics within a 100 m front crawl event [e.g., (24)]. The swimmers were not able to maintain velocity denoting inter-lap reductions in stroke rate and a maintenance in stroke length. The 100 m front crawl event is commonly classified as an extreme-intensity effort (25) where fatigue is expected to show up in the upper body muscles, decreasing the swimmers’ power-producing capacity and changing kinematics (26).

The kinematics of each stroke cycle relies on a relationship between stroke rate and stroke length with different combinations for velocity or efficiency maintenance (10, 19). Previous studies showed that decreases in stroke rate were linearly related to decreases in velocity (27) being a typical behavior of swimmers competing at an international level in a 100 m front crawl race (28). Still, the capacity to maintain stroke length should not be discarded as a skill to mitigate the loss of efficiency from the first to the second part of the 100 m effort.

The ability to maintain swimming velocity (29), or present a more stable kinematic profile (30) are decisive factors for optimizing performance and defining the swimmers’ chance to be in a final or winning a medal. The inter-lap assessment arises here as an approach that allows understanding the swimmers’ variance in a given variable across multiple laps (8). It can be used to identify patterns of performance over time and to evaluate how different factors (such as fatigue, training interventions, or environmental conditions) affect performance. In the current study, the inter-lap changes revealed negative values near 5%–6%, which is not so far from studies with swimmers participating in European Championships (28). Although negative values should be expected during inter-lap analysis, those are not necessarily indicative of poor performance or a negative outcome. Instead, they should be interpreted in the context of the overall performance goals and the specific factors being evaluated. In some cases, negative values may be expected or even desirable (such as when deliberately altering technique as part of a race strategy).

Short-distance swimming specialists, such as sprinters, rely heavily on explosive power and strength to generate the velocity and the in-water force needed to perform at their best, requiring high levels of anaerobic power and muscular endurance (31). Typically, those are able to generate high levels of force and power during tethered swimming tests, indicating their superior strength potential in the water (32). The swimmers of the present study exhibited peak force values of 375 N and mean force values of 121 N, which is not so far from what was reported in the literature [e.g., (33)]. The peak force values during a tethered swimming test already showed a great association with the 100 m front crawl velocity (5). But, the association between the changing behavior within a race and the tethered swimming measures was unknown. In the current study, the swimmers who displayed greater peak force values were the ones who showed a lower velocity variation. It means that those are fitter and better-conditioned swimmers, and capable to maintain their technique for a longer set of the race, even as fatigue sets in.

The relationship between inter-lap kinematics, velocity and peak force is likely complex and multifaceted, and there may be other factors that contribute to this association as well. While the training load may change during the specific stages of a season (34), the conditioning status of this type of swimmers can be monitored through the peak force retrieved in a tethered swimming test. Even so, more research will be needed to fully understand the mechanisms underlying this relationship and to increase practical strategies for swimmers and coaches to optimize their performance. Conducting research about associations between tethered swimming measures and inter-lap adaptations in the remaining swimming strokes or distances should be a priority in the future. Plus, the behavior of the in-water forces must be understood according to the time spent by each swimmer in performing a certain distance.

## 5. Conclusions

It can be concluded that the swimmers who exhibit higher peak force values are those who show a more stable front crawl velocity during a 100 m front crawl effort, even if both pacing and kinematics fall from the first to the second 50 m sections. Coaches can monitor their fastest swimmers’ strength on the progression of peak forces over the season using the tethered swimming method.

## Data Availability

The datasets presented in this article are not readily available because these are data from a larger project that still is under course. Requests to access the datasets should be directed to Mário Costa, mjcosta@fade.up.pt.
